# 1946. *In Vitro* Activity of Ceftobiprole against *Staphylococcus aureus* Bacteremia Isolates from the United States (2018–2020)

**DOI:** 10.1093/ofid/ofad500.100

**Published:** 2023-11-27

**Authors:** Leonard R Duncan, Mariana Castanheira, Jennifer Smart, Mark E Jones, Rodrigo E Mendes

**Affiliations:** JMI Laboratories, North Liberty, Iowa; JMI Laboratories, North Liberty, Iowa; Basilea Pharmaceutica International Ltd, Allschwil, Basel-Landschaft, Switzerland; Basilea Pharmaceutica International Ltd., Allschwil, Switzerland, Allschwil,, Basel-Landschaft, Switzerland; JMI Laboratories, North Liberty, Iowa

## Abstract

**Background:**

Ceftobiprole (BPR) is an advanced-generation cephalosporin approved in Europe and many non-European countries for the treatment of community-acquired pneumonia and non–ventilator-associated hospital-acquired pneumonia in adults. A Phase 3 clinical trial (NCT03138733) was recently completed with BPR for the treatment of *Staphylococcus aureus* (SA) bacteremia, including infective endocarditis. This study evaluated the activity of BPR and comparators against recent SA isolates causing bloodstream infections (BSI) in patients hospitalized at medical centers in the United States (US).

**Methods:**

A total of 1,946 SA isolates from 32 US medical centers (2018–2020) were collected from patients with BSI. 53 isolates from endocarditis patients were included. Isolates were tested for antimicrobial susceptibility using the Clinical and Laboratory Standards Institute (CLSI) broth microdilution method. MIC interpretations for BPR and comparators utilized European Committee on Antimicrobial Susceptibility Testing (EUCAST) or CLSI criteria, respectively. Multidrug-resistant (MDR) isolates were non-susceptible to ≥ 3 of the following drugs without regard to oxacillin resistance: clindamycin, daptomycin, erthryomycin, gentamicin, levofloxacin, tetracycline, and trimethoprim-sulfamethoxazole. No isolates were resistant to linezolid, tigecycline, or vancomycin.

**Results:**

BPR inhibited 99.5% of all SA at ≤ 2 mg/L, which is the EUCAST susceptibility breakpoint (MIC_50/90_, 0.5/2 mg/L). The MIC_50/90_ values were stable over all 3 surveillance years and identical to previously reported values from analogous isolates collected in 2016–2017. BPR activity was maintained against the methicillin-resistant SA (MRSA) subset (41% of all SA; 98.8% susceptible). All isolates from endocarditis patients were susceptible to BPR. 10 MRSA isolates (0.5%) were resistant to BPR with MIC values of 4 mg/L. Of 288 MDR isolates, 96.9% remained susceptible to BPR, while only 78.8% of the same isolate set was susceptible to ceftaroline.

**Conclusion:**

These *in vitro* results extend previous surveillance data that suggest that BPR represents a potential option for treating BSI and endocarditis caused by SA in US hospitals, including MRSA and MDR isolates.

**Disclosures:**

**Leonard R. Duncan, PhD**, AbbVie: Grant/Research Support|Basilea: Grant/Research Support|CorMedix: Grant/Research Support|Melinta: Grant/Research Support|Pfizer: Grant/Research Support **Mariana Castanheira, PhD**, AbbVie: Grant/Research Support|Basilea: Grant/Research Support|bioMerieux: Grant/Research Support|Cipla: Grant/Research Support|CorMedix: Grant/Research Support|Entasis: Grant/Research Support|Melinta: Grant/Research Support|Paratek: Grant/Research Support|Pfizer: Grant/Research Support|Shionogi: Grant/Research Support **Jennifer Smart, PhD**, Basilea Pharmaceutica International Ltd, Allschwil, Switzerland: Stocks/Bonds **Mark E. Jones, PhD**, Astellas Pharma Global Development, Inc: Support for the present publication|Basilea Pharmaceutica International Ltd: Employee of Basilea Pharmaceutica International Ltd|Basilea Pharmaceutica International Ltd: Stocks/Bonds **Rodrigo E. Mendes, PhD**, AbbVie: Grant/Research Support|Basilea: Grant/Research Support|Cipla: Grant/Research Support|Entasis: Grant/Research Support|GSK: Grant/Research Support|Paratek: Grant/Research Support|Pfizer: Grant/Research Support|Shionogi: Grant/Research Support

